# Small but strong: Pivotal roles and potential applications of snoRNAs in hematopoietic malignancies

**DOI:** 10.3389/fonc.2022.939465

**Published:** 2022-08-12

**Authors:** Jian Dong, Hui Wang, Zhaoru Zhang, Lin Yang, Xinyue Qian, Wenchang Qian, Yingli Han, He Huang, Pengxu Qian

**Affiliations:** ^1^ Center of Stem Cell and Regenerative Medicine, and Bone Marrow Transplantation Center of the First Affiliated Hospital, Zhejiang University School of Medicine, Hangzhou, China; ^2^ Liangzhu Laboratory, Zhejiang University Medical Center, Hangzhou, China; ^3^ Institute of Hematology, Zhejiang University & Zhejiang Engineering Laboratory for Stem Cell and Immunotherapy, Hangzhou, China; ^4^ Bone Marrow Transplantation Center, The First Affiliated Hospital, Zhejiang University School of Medicine, Hangzhou, China

**Keywords:** small nucleolar RNA, rRNA modification, 2’-O-methylation, hematological malignancies, epigenetics

## Abstract

Small nucleolar RNAs (snoRNAs) belong to a family of noncoding RNAs that are 60-300 nucleotides in length, and they are classified into two classes according to their structure and function: C/D box snoRNAs, playing an essential role in 2’-O-methylation modification on ribosomal RNA; H/ACA box snoRNAs, involved in the pseudouridylation of rRNA. SnoRNAs with unclear functions, no predictable targets, and unusual subcellular locations are called orphan snoRNAs. Recent studies have revealed abnormal expression and demonstrated the pivotal roles of snoRNAs and their host genes in various types of hematological malignancies. This review discusses recent discoveries concerning snoRNAs in a variety of hematological malignancies, including multiple myeloma, lymphoma and leukemia, and sheds light on the application of snoRNAs as diagnostic and prognostic markers as well as therapeutic targets of hematological malignancies in the future.

## Introduction

Hematopoiesis is the essential physiological process of maintaining the blood system, which relies on a small population of cells that can differentiate into blood progenitor cells and self-renew, namely, hematopoietic stem cells (HSCs). HSCs first differentiate into diverse lineage-restricted progenitors and then turn into various mature lineage cells. HSCs are maintained in a quiescent state coordinated by a complicated network. Some reports indicate that HSCs are characterized by lower protein synthesis efficiency (1, 2). When protein synthesis is disrupted in HSCs, HSCs exit homeostasis and exhibit certain features akin to malignant cells. In this context, translational regulation by epitranscriptomic modification of rRNA and tRNA may have a crucial effect on hematopoiesis.

In 2020, over 1.3 million new cases of hematological malignancy (including myeloma, leukemia, and lymphoma) were diagnosed worldwide (3), accounting for 6.6% of all newly diagnosed cancer patients, while 711,840 hematological disease-related deaths accounted for 7.1% of total cancer-related casualties. Hematological malignancies are characterized by dysregulation of hematopoiesis, which can occur in bone marrow, spleen, lymph nodes, and other tissues (4–10). The primary syndromes of patients suffering from these diseases are anemia, infection, and bone marrow failure. Conventional therapeutic approaches, including radiotherapy and chemotherapy, are practical for patients in the early stages. Moreover, hematopoietic stem cell transplantation (HSCT) is the standard choice for late-stage patients who are unresponsive to chemo/radio-therapies (8). Nevertheless, relapse is still the major obstacle faced with all therapies. With the innovation of therapeutic approaches, scientists have developed chimeric antigen receptor T cells (CAR-T cells) (11), new drugs for immune regulation and high-efficiency radiotherapy as tools in clinical treatment, which significantly increase the clinical outcome (12, 13). However, we still do not know much about the mechanisms underlying hematological malignancies. Thus, elucidation of novel mechanisms would facilitate the treatment of these diseases.

Early research on small nucleolar RNAs (snoRNAs) started in the late 1960s (14), and this type of small RNA was first discovered as a factor associated with the processing of ribosomal RNA (rRNA) (15, 16). They are encoded in host genes with independent promoters or in introns of genes without promoters. Transcription of this type of molecule is predominantly driven by RNA polymerase II, while others are occasionally driven by specific elements of pol III (17). SnoRNAs consist of 60-300 nucleotides (nts) and are mainly divided into C/D box snoRNAs and H/ACA box snoRNAs. Some snoRNAs have no predictable target or clear function and are called orphan snoRNAs. Two conserved structures characterize C/D box snoRNAs: the C box (RUGAUGA) and D box (CUGA) (18), which are located at the 5’ and 3’ ends of RNA, respectively. There is an essential element for assembling a small nucleolar ribonucleoprotein (snoRNP) complex called kink-turn, which is a structure of stem-bulge-stem binding to p15.5KD protein, one of the core proteins of C/D box snoRNP. The core components of C/D box snoRNAs include Nop56 (19), Nop58/Nop5p (20), p15.5KD/Snu13p (21) and fibrillarin (FBL) (22). FBL is a methyltransferase responsible for site-specific 2’O-methylation of rRNA and small nuclear RNAs (snRNAs). Most C/D box snoRNAs contain C’/D’ box sequences, one or two bases different from the C/D box. The upstream 5’ sequence of the D or D’ box is complementary to target RNAs, guiding the accurate modification of the fifth base of the upstream D or D’ box motif. C boxes are necessary in the formation of snoRNPs (23), and D boxes are foundational in site selection (16). A helix structure is formed by the binding of snoRNA and target RNA during 2’-O-methylation, and this type of modification is important in maintaining the normal structure of rRNA, protecting it from hydrolysis, and influencing translation fidelity and ribosome biogenesis. Recent evidence revealed that 2’-O-methylation affects translation at internal ribosome entry sites (IRES) (24). H/ACA box snoRNAs contain Box H (ANANNA, N represent any one of four nucleotides) and box ACA motifs (25) and are characterized by a unique secondary structure of a “hairpin-hinge-hairpin-tail” (26). There is an internal loop located in one or both hairpins with a sequence complementary to the target RNA, important to the location of the pseudouridylation site; the modified base is usually located 14-16 nt downstream of the H box or ACA box (27). The core proteins of H/ACA snoRNP include DKC1 (pseudouridine synthase) (28), Nop10 (29), GAR1 (30), and Nhp2 (29). Pseudouridine of RNA has several advantages compared with unmodified uridine, such as folding of rRNA (31), transcript stability (32) and interaction between RNAs and proteins (33). Structural changes were observed with loss of rRNA pseudouridine, and the peptidyl transferase center (PTC) was also affected (34). Hence, pseudouridine may play an important role in the stabilization of the ribosome subunit. Moreover, impairment of the cells lacking five snoRNAs (−6Ψs) was more severe than that observed for loss of the Ψ2919 guide snoRNA alone, suggesting the possibility of synergy between H/ACA snoRNAs (34). SnoRNAs are mainly located in the nucleolus and involved in many important biological processes, such as development and carcinogenesis, through modification of rRNA and snRNA. However, the roles and mechanisms of snoRNAs in various biological processes warrant further exploration.

**Figure 1:**
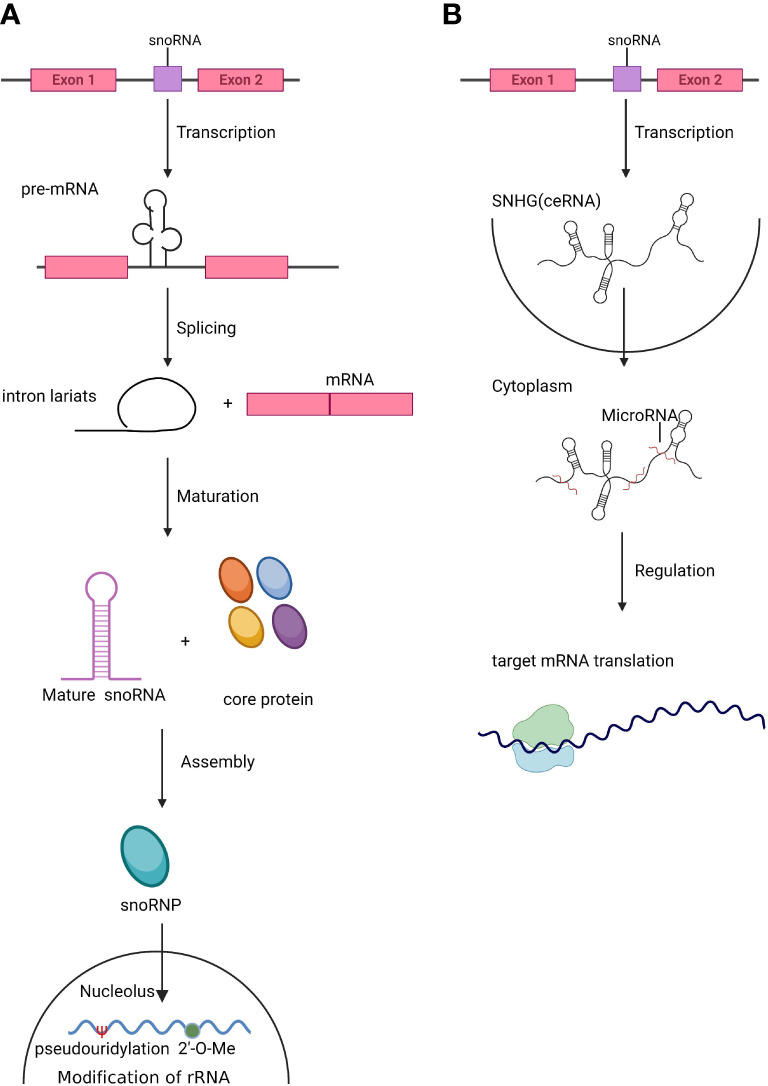
The biogenesis of snoRNAs and SNHGs. **(A).** Small nucleolar RNAs (snoRNAs) are mainly located in introns. After splicing and maturation, mature snoRNAs are exported to the nucleolus, where they assist in the modification and processing of rRNA and snRNA. **(B).** Small nucleolar RNA host genes (SNHGs) are transcribed and act as competing endogenous RNAs (ceRNAs) by antagonizing miRNAs and modulating the translation of target genes.

As one of the most abundant small noncoding RNAs, snoRNAs affect many critical cellular processes by regulating rRNA modification, processing, and ribosome function. The most recognized role of snoRNAs is the site-specific modification of pre-rRNA and snRNA to facilitate the maturation of rRNA (**Figure 1A**), thus regulating the process of translation, and their host gene can also act as competing endogenous RNA (ceRNA) in modulation of translation (**Figure 1B**). Dysregulation of snoRNAs may cause many diseases. In the intricate network that maintains hematopoiesis, HSCs have a unique role in both self-renewal and differentiation; progenitors undertake the replenishment of hematopoietic cells, while HSCs remain in a relatively quiescent state (35). If the balance between self-renewal and differentiation is disrupted, insufficient differentiation and abnormal self-renewal of HSCs eventually cause hematological diseases (36). The network of HSC regulation comprise a number of factors and pathways. Regulation of ribosome function is a peculiarly pivotal factor linked to HSC self-renewal and differentiation (1). Recent research revealed that some mRNAs necessary in HSC maintenance have higher translation efficiency in the context of lower levels of protein synthesis in HSCs (2). In this regard, both rRNA and tRNA are key factors in the control of translational efficiency and protein fidelity. Thus, the chemical modifications of these RNAs, guided by snoRNAs, play crucial roles in maintaining HSC homeostasis and stress responses (37).

High fidelity is needed in the maintenance of HSCs, and defects in translation fidelity control may impair self-renewal. For instance, c-Myc is a well-known gene that can cause defects in the self-renewal of HSCs (38), and it is notable that c-Myc has been reported to be a regulator of snoRNA biogenesis (39). Subsequent alterations in protein synthesis disrupt normal hematopoiesis, ultimately leading to hematological malignancies. In normal hematopoietic differentiation, the expression of snoRNA is cell type specific; snoRNAs located in the Dlk-Dio3 locus, imprinted region on mouse chromosome 12 (40), have the highest expression in long-term HSCs and exhibit a gradual decrease with HSC differentiation (41). Furthermore, it is well known that mutation of the transcription factor (TF) RUNX1/AML1 is observed frequently in patients with myelodysplastic syndrome and leukemia, and the mutation is linked to the expression of rRNA and ribosomal proteins (42). After years of clonal expansion, cells with such mutations could outcompete normal hematopoietic stem and progenitor cells (HSPCs) in the bone marrow, resulting in hematological malignancies. The latest studies have found that the fusion protein AML1-ETO can promote leukemic cell self-renewal (43). Thus, these studies suggest that snoRNAs might play pivotal roles in normal and malignant hematopoiesis. Since few studies have elucidated the roles of snoRNAs in HSCs, we will mainly summarize the recent literature on the roles of snoRNAs in different hematopoietic diseases.

## Roles of snoRNAs in hematopoietic malignancies

### snoRNAs in multiple myeloma

As the second most common malignancy in the hematological system, the median age of patients with multiple myeloma (MM) is 62 for men and 61 for women. Patients often suffer from anemia, renal failure, and cortical bone destruction (6). Unfortunately, this disease is currently incurable. The commonly used therapeutic tactics include high-dose chemotherapy and autologous stem cell transplantation. In addition, some novel treatments have been developed, such as immunomodulatory drugs, proteasome inhibitors, and monoclonal antibody-based therapies. To some extent, these therapeutic drugs benefit patients, but a high relapse rate is still the main problem. In recently published research, the authors found that some snoRNAs may play an essential role in the progression of MM.

**Figure 2:**
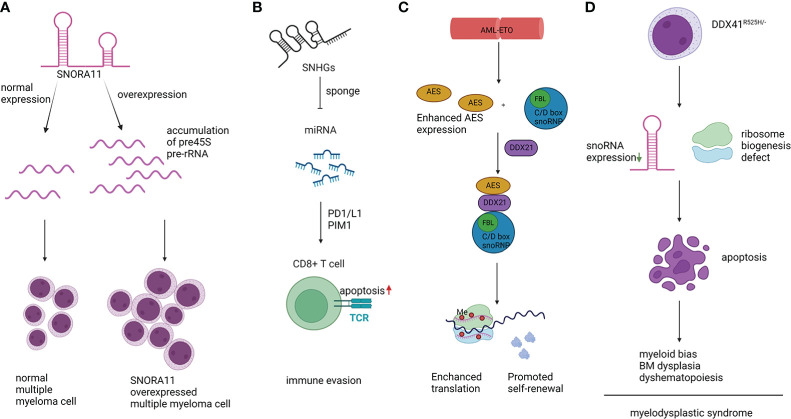
Representative mechanisms of snoRNAs in hematopoietic malignancies. **(A).** snoRNA ACA11 resulted in accumulated 45S pre-rRNA, promoted proliferation, and increased cell size. **(B).** SNHG sponged miRNAs and promoted myeloma cell immune evasion. **(C).** AML1-ETO protein promoted self-renewal of AML cells depending on the interaction between C/D box snoRNA and DDX21. **(D).** DDX41^R525H/-^ caused dysregulation in hematopoiesis, downregulation of snoRNA expression, and defects in ribosome biogenesis.

Orphan snoRNA ACA11, encoded within an intron of WHSC1 (also known as MMSET) (44), was overexpressed in MM patients with a t(4;14) chromosomal translocation (45). Overexpression of ACA11 led to upregulated ribosome biogenesis, protein synthesis, and larger cell size. The accelerated level of pre-45S rRNA transcription, which is the rate-limiting step of rRNA processing, resulted in the increased level of 45S pre-rRNA in ACA11-overexpressing MM cells. Overall, ACA11 overexpression in MM cells upregulated ribosome biogenesis in a ROS-dependent manner. As a result, MM cells had a higher proliferation rate, and ACA11 accelerated the progression of MM disease (**Figure 2A**). The above result identified ACA11 as a key factor in the pathogenesis of MM, implying therapeutic potential for patients with t(4;14) mutation.

Moreover, SNORD115 and SNORD116 were aberrantly expressed in one subgroup of MM patients with low-moderate levels of cyclin D1 (CCND1) without any primary immunoglobulin H (IgH) translocation or hyperdiploidy (HD) (46). The SNORD115 & SNORD116 families are located at 15q11, which is an imprinting region. In patients with MM with translocation/cyclin D4 (TC4), SCARNA22 was highly expressed. Moreover, six H/ACA boxes (SNORA40, -74A, -64, -23, -22 and -68) and five C/D box snoRNAs (SNORD24, -36B, -63, -36C and -D95) were overexpressed in the HD setting versus non-HD cases. These studies imply that snoRNAs could be used as pathological markers for the diagnosis of specific subtypes of MM (46). Although the functions and mechanisms of snoRNAs in MM are still elusive, some studies have reported their potential in disease diagnosis and prognosis. In a snoRNA profiling study of 55 MM cases, 8 secondary plasma cell leukemia cases (sPCL) and 4 healthy controls (46), SNORD32A and SNORA42 were found to be downregulated in sPCL. SNORD32A was also reported to be involved in the noncanonical process of oxidative and endoplasmic reticulum stress-induced response pathways *in vitro* and *in vivo* (46). There is a low correlation between hematopoietic malignancy and SNORA42 expression, but the SNORA42 region, which is located on chromosome 1q22, is frequently amplified in plasma cell dyscrasias. In addition, SNORA42 itself was found to be a putative oncogene in non-small cell lung cancer (47).

SNHG 18 is a long noncoding (LNC) RNA and is also the host gene of SNORD123. SNHG18 had a higher expression level in MM bone marrow samples. It is involved in cell migration and adhesion by interacting with semaphorin 5A (SEMA5A), which is also overexpressed in some MM bone marrow samples (48). The correlation between SNHG18 expression and MM prognosis was discovered according to multiple criteria, including International Staging System (ISS) classification, Revised International Staging System (R-ISS) classification, Mayo Clinic Risk Stratification for Multiple Myeloma (mSMART), and other standards for multiple myeloma classification. The patients with high expression of both SNHG18 and SNORD123 had shorter OS times than those with low expression of two genes or high expression of only one gene.

### snoRNAs in lymphoma

Lymphoma is named by its organ of origin, and there are two main categories: non-Hodgkin lymphoma (NHL) and Hodgkin lymphoma. Symptoms include fever, night sweating, unintended weight loss, and enlarged lymph nodes. Usually, patients have no feeling of pain at the primary lymphoma stage, but with the progression of the disease, they suffer from bone pain, itching, and anemia (7, 9). Lymphoma is usually diagnosed by biopsy of bone marrow and lymph nodes, and treatment (HSCT, radio/chemotherapy, etc.) will be given to patients based on the invasion and growth rate. With the highest incidence, lymphoma is the leading cause of mortality among hematological malignancies, and the involvement of snoRNAs in lymphoma is still elusive.

Previous studies reported that snoRNAs play critical roles in lymphoma. In diffuse large B-cell lymphoma (DLBCL), overexpression of snoRNA host gene (SNHG)12, the host gene of SNORA44, SNORA61, and SNOR16B/A family, was correlated with poor prognosis and accelerated tumorigenesis by sponging miR-195 (49). The authors also investigated the effect of SNHG12 *in vitro* and *in vivo*. They found that overexpression of SNH12 promoted cell growth, migration, and invasion of DLBCL cells, which was also verified in a xenograft mouse model. Lina Zhao and colleagues found the upregulation of SHNG14 in DLBCL patient samples by microarray analysis. When SNHG14 was knocked down in FARAGE and U2932, two DLBCL cell lines, cell viability and colony formation ability were impaired. Invasive ability and epithelial-to-mesenchymal transition (EMT) were also suppressed (50). Mechanistically, SNHG14 acted as a sponge of miR-5590-3p, lowering its expression and triggering apoptosis in CD8+ T cells through PD-1/PD-L1. This effect helped DLBCL cells escape the immune response. Moreover, ZEB1, the target of miR-5590-3p, was also upregulated in DLBCL cells and was responsible for the overexpression of PD-1/L1 and contributed to immune evasion in the tumor microenvironment (**Figure 2B**). Similarly, Yuyang Tian and colleagues found that the interaction between SNHG14 and miR-152-3p promoted tumor progression and immune evasion by inhibiting cytotoxic T lymphocytes (CTLs) (51). In addition, Qiaojuan Zhu et al. found that SNHG16 promoted DLBCL cell proliferation through the miR-497-5p/PIM1 axis. SNHG16 functions as a competing endogenous RNA (ceRNA) in DLBCL by interacting with miR-497-5p, a tumor suppressor in DLBCL (52). In addition, in the chromosome breakpoint t(3,6)(q27;q15) in the DLB cell line (53), a new snoRNA host gene family, U50HG, was reported. Transcriptional dysregulation of BCL6 was associated with 3q27 in B-cell lymphoma by promoter substitution (54). Although there was no direct evidence that U50 snoRNA was involved in this process, considering its function of guiding 2’-O-methylation of C2849 and G2864 of 28S rRNA (16), it is plausible that U50 snoRNA was also involved in the process of lymphoma progression, at least in part. Furthermore, t(3,6)(q27;q15) influenced the expression of U50 snoRNA and partially affected the biogenesis or activity of ribosomes by impairing the chemical modification of C2849/G2864 (53). Through specific deletion of mU50, the authors observed an increase in abnormal events in mouse lymphocytes, including differentially expressed heat shock proteins (55).

Peripheral T-cell lymphoma (PTCL), a rare type of NHL, is associated with poor clinical outcomes. The snoRNA expression signature can be used as a novel biomarker for a more precise subdivision of PTCLs. A set of 30 snoRNA signatures was discovered with robust expression in anaplastic large cell lymphoma (ALCL) and can be used as a diagnostic marker to discriminate ALCL from non-ALCL patients. Among these snoRNAs, U75 snoRNA is the most potent classifier (56). Furthermore, U3 snoRNA can distinguish ALK+ from ALK- ALCL patients as a single marker. AITC/PTCL-NOS can be further divided into three subgroups using the snoRNA expression signature, but no significant snoRNA expression was discovered between AITC and PTCL-NOS patients. In addition, in AITL patients, overexpression of HBII-239, U59B, and U90 was correlated with a better prognosis, represented by prolonged overall survival (OS) and progression-free survival (PFS). According to the snoRNA profile, eleven snoRNAs were significantly upregulated in PTCL-NOS patients, with HB II-239, HBII-438A, and U80 being the most prominent, predicting a better prognosis and OS. HBII-239 was the most powerful among these markers (56), benefiting PFS and OS simultaneously. One HB II-239 processed microRNA (miRNA), miR-768-3p, showed distinct expression between the groups with different outcomes. Furthermore, Zhu et al. found that SNHG12 was upregulated by c-Myc in natural killer/T-cell lymphoma (NKTCL) and influenced proliferation and drug sensitivity, which may explain why NKTCL cells are highly resistant to chemotherapy and multiple drugs (57).

### snoRNAs in myeloid leukemia

Acute myeloid leukemia (AML) is a hematological malignancy characterized by the uncontrollable expansion of leukemic stem cells and abnormally differentiated hematopoietic cells (4). In acute myeloid leukemia with chromatin t (8;21) translocation, AML1-ETO enhanced C/D box snoRNP and rRNA 2’-O-methylation and facilitated the interaction between amino-terminal enhancers of split (AES) and RNA helicase DDX21, which eventually hastened cell proliferation *in vitro* and promoted leukemogenesis *in vivo* (43). In addition, the authors found that snoRNAs, including SNORD34, SNORD35A, and SNORD43, played an important role. In Kasumi-1 cells, knocking down these snoRNAs led to decreased rRNA 2’-O-methylation, impaired protein synthesis, and reduced clonogenic growth (**Figure 2C**). Other oncogenes, such as MYC and MLL-AF9, can also upregulate the expression level of snoRNA. The above phenomenon indicates that snoRNAs are involved in the intricate network of AML modulation under the control of oncogenes. The detailed mechanism is still elusive, but dysregulation of translation efficiency and fidelity influenced by changes in 2’-O-methylation has been observed. Accelerated protein synthesis is a pivotal factor in the process of cancer cell proliferation. Similarly, another study reported that SNORD42A was involved in AML progression by affecting site-specific methylation of 18S rRNA. Deletion of SNORD42A significantly delayed leukemogenesis *in vivo* and impaired self-renewal of HSCs in an HSCT assay (58). Intriguingly, SNORD42A was reported to bind to nucleophosmin 1 (NPM1), which is a well-known nucleocytoplasmic shuttling protein enriched in the nucleolus. Mutation of NPM1 is one of most common events observed in AML, and over 30% of AML patients harbor frame shifts in the region encoding the C-terminus of NPM1. These variants result in cytoplasmic resident of NPM1 (NPMc) (59). Reduced 2’-O-meth modification levels can be observed in samples from patients with NPMc mutations or AML cell lines with this mutation. In K562, a cell line of AML, decreased colony formation ability was observed after inactivation of SNORD15, SNORD47, SNORD104, and knockout of SNORD15, SNORD52, SNORD58 promoted erythroid differentiation of cells. SnoRNA is the most abundant RNA bound to NPM1, and a significant reduction in 2’-O-methylation was observed after knockout of NPM1, whereas the global translation efficiency was not altered (60). However, changes in the translation levels of some specific proteins were discovered, such as Cdkn1b, Xiap, and Vegf, indicating their influence on translation. The above results indicated the significance of NPM1-regulated ribosomal efficiency by direct binding to C/D box snoRNAs, thus influencing the growth and differentiation of leukemia cells (60).

Moreover, Shi et al. found that the PTEN/PI3K/AKT axis, modulated by SNHG16 in AML, could promote the proliferation and migration of leukemia cells (61). Mechanistically, SNHG16 interacted with miR-19, and SNHG16 knockdown suppressed proliferation and induced apoptosis in AML cells. Similarly, SNHG5 has been reported to contribute to angiogenesis in AML through the miR-256b/CTGF/VEGFA axis. SNHG5 has a high expression level in AML cells and is regulated by the Yin Yang1 (YY1) protein, which can directly bind to the SNHG5 promoter (62). SNHG1 was also reported to modulate leukemia progression and indicate poor prognosis in AML patients (63). Another signaling pathway involves miR-489-3p/SOX12/Wnt/β-catenin, which is modulated by SNHG1 and contributes to AML progression (64). SNHG4 was found to regulate the proliferation of AML cells *via* the miR-10a/PTEN axis. SNHG4 was downregulated in AML patients and reduced the expression of miR-10a, which led to increased expression of PTEN and inhibited proliferation of AML cells (65).

In acute promyelocytic leukemia (APL), the SNORD112-114 cluster was reported to be upregulated (41). Compared with CD33^+^ cells from healthy donors, snoRNAs in the SNORD112-114 cluster were downregulated in AML samples, whereas they were overexpressed in APL samples carrying PML-RARα_BCR1 translocations. The overexpression of SNORD114-1 led to reduced expression of Rb protein, accelerated the cell cycle, and finally promoted disease progression. In addition, T Liuksiala et al. also found that overexpression of SNORD114-3 served as a new biomarker in APL patients by compiling hematological gene expression data (66).

In chronic myeloid leukemia (CML), SNHG5 regulates the proliferation, differentiation, and apoptosis of leukemia cells by inhibiting methylation of the death receptor 4 (DR4) gene (67). Inhibition of SHNG5 resulted in increased apoptosis and differentiation of leukemia cells and suppressed cell proliferation. In another study, Dan Wang et al. reported that SNHG5 knockdown could enhance the sensitivity of AML cells to chemotherapy through the miR-32/DNAJB9 axis (68).

### snoRNAs in lymphocytic leukemia

Chronic lymphocytic leukemia (CLL), one of the most common chronic leukemias in adult patients, is caused by a disorder in lymphocytes (8). Domenica Ronchetti et al. examined snoRNA expression in 211 CLL patients and found extensive downregulation of snoRNA in patients with poor prognosis. For example, SNORA70F and its host gene COBLL1 were significantly downregulated (69), SNORA31 and its host gene TPT1 both showed reduced expression, and TPT1 is well known to influence stemness by regulating the function of TP53 (70). Moreover, Domenica Ronchetti et al. found that lower expression of SNORA74 and SNORD116-18 distinguished CLL patients with better clinical outcomes. The features of snoRNAs were independent of common mutation hot spots and cytogenetic markers, such as ZAP-70 and CD38. Thus, the snoRNA signature can act as a novel biomarker to refine the classification of CLL patients.

Laure Berquet et al. found that the expression of a set of 20 snoRNAs was associated with treatment-free survival (TFS) in CLL patients with IGHV mutation. The median TFS of patients with high snoRNA expression levels was 32 months, in contrast to 144 months in the lower expression cohort (71). Furthermore, Gurvinder Kaur et al. reported altered expression of several miRNAs in CLL patients compared with healthy donors. miR-763 is located on chromosome 22 of SNORD43 (72). These aberrantly expressed miRNAs target crucial genes in CLL, such as ATM and TP53, which are involved in important pathways, such as RNA transport, the cell cycle, mTOR and p53 signaling.

In T-cell acute lymphocytic leukemia (T-ALL) patients with poor prognosis, deletion of 6q was one of the most common chromosomal abnormalities. Stéphanie Gachet et al. reported that SHNG5 was involved in T-ALL progression by cooperating with SYNCRIP and had potential for therapeutic intervention (73). The combined haploinsufficiency of SHNG5 and SYNCRIP significantly accelerated leukemogenesis of Tal1/Lmo1/Notch1-induced T-ALL in a mouse model. In human T-ALL cells, ribosome and mitochondrial dysfunction were observed along with deletion of the SYNCRIP-SNHG5 region, which developed leukemia more efficiently in the competitive engraftment experiment and limit-dilution experiment, indicating increased malignancy and enhanced leukemia-initiating cell (LIC) activity.

Acute lymphoblastic leukemia (ALL) patients comprised 25% of cancer patients under 15 years old. SNHG16 was found to be upregulated in ALL cells. Tianxin Yang et al. reported that SNHG16 acted as an oncogene in ALL. Downregulation of SNHG16 inhibited leukemogenesis, migration, and proliferation of ALL cells *in vitro* and *in vivo* through an epigenetic mechanism (74). Mechanistically, miR-124-3p acted downstream of SNHG16, which could reverse the effect of SNHG16 downregulation in ALL and enhance the migration ability. Kaisa J. Teittinen et al. found a distinct snoRNA pattern between T-ALL and pre-B- ALL by massive parallel sequencing (75). This expression pattern comprised four box C/D snoRNAs (SNORD24, SNORD44, SNORD82, SNORD105) and two scaRNAs (scaRNA6 and scaRNA9). Moreover, in ERG-related childhood B-cell precursor lymphoblastic leukemia (BCP-ALL), characterized by aberrant expression of ERG-related genes and deletion of ERG, SNORD116 was found to be upregulated (76). A set of snoRNAs (SNORD64, SNORD107, SNORD109A, SNORD116) were found to be upregulated in ERG-related BCP-ALL compared with ERG-nonrelated BCP-ALL samples. Furthermore, these snoRNAs are located in the chromosome 15q11.2 region, which is also involved in the pathogenesis of Prader-Will Syndrome (PWS) (77). Meanwhile, aberrant expression of the rRNA methylation complex was also observed in patients with BCP-ALL. The core proteins of C/D box snoRNA, FBL and NOP56 were upregulated by c-Myc (43). In addition, SNORD35B, SNORD46, and SNORD65 were reported to be upregulated in relapsed BCP-ALL patients compared with patients without relapse (78). Together, these studies indicate the potential of snoRNAs in the classification of different leukemic subtypes.

### SnoRNAs in myelodysplastic syndrome

Myelodysplastic syndrome (MDS) is a clonal disorder resulting from abnormal HSCs. MDS patients have typical symptoms of cytopenia in both bone marrow and peripheral blood caused by ineffective hematopoiesis, and blast cells can be detected in blood cells with evident dysplastic morphology. A recent elegant study reported that SNORA7A, SNORA16A, and SNORA70 were downregulated in DDX41-deficient cells, which impaired cell proliferation and differentiation and led to cell cycle arrest (79). Moreover, DDX41 deletion increased the abundance of 45S rRNA and decreased the abundance of other subunits of rRNA, which eventually led to defects in ribosome biogenesis and reduced protein synthesis. These results suggest that the commonly observed mutation of DDX41 in MDS influenced protein synthesis by affecting the snoRNA-ribosome axis (**Figure 2D**).

## Diagnostic, prognostic and therapeutic potential of snoRNAs in hematopoietic malignancies

**Figure 3:**
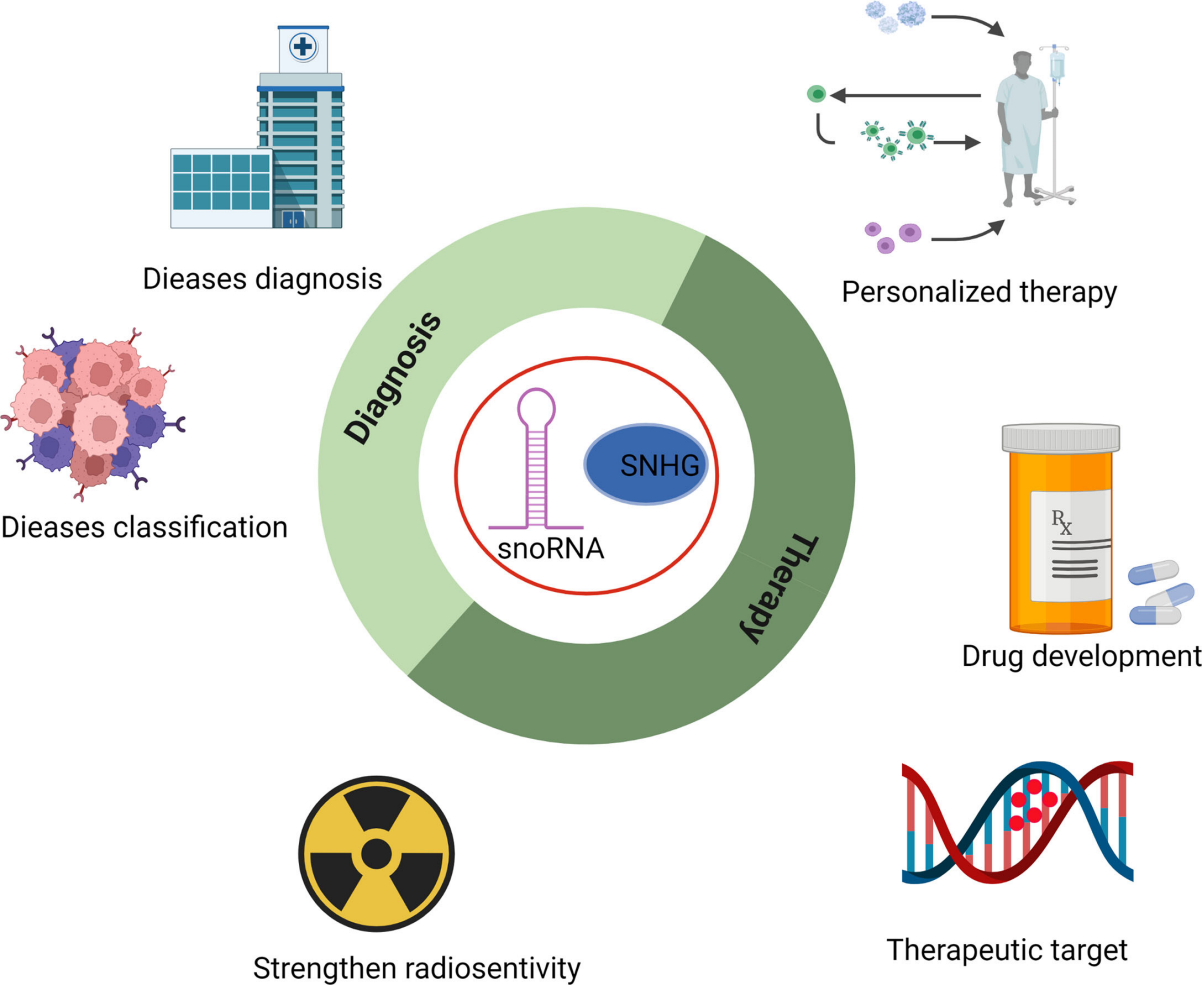
Potential application of snoRNAs in the diagnosis, classification, strengthening radiosensitivity, drug development, and personalized therapy of hematopoietic malignancies.

Based on the findings mentioned above, snoRNAs and their host genes play essential roles in the progression of several hematological malignancies and may serve as novel candidates for disease prevention and treatment (**Figure 3**). For example, enhanced formation of C/D box RNP is required for leukemogenesis driven by AML1-ETO, the chimeric protein resulting from t (8;21) translocation (43). Deleting SNORD14D impaired colony formation ability, and depletion of SNORD14D, SNORD34, SNORD35A, or SNORD43 decreased cell size and reduced the protein synthesis rate. Moreover, SNORD42A has been reported as a regulator of 2’-O-methylation of the 40S subunit. Overexpression of SNORD42A increased the translation efficiency of various oncogenes and influenced proliferation, indicating the potential of therapeutic application in AML (58). SNHG12, the host gene of multiple snoRNAs, promoted the evasion and migration of DLBCL cells, and knockdown of SNHG12 delayed tumorigenesis (49). All these discoveries indicated that snoRNAs are feasible targets for the diagnosis, prognosis, and treatment of leukemia, and rectifying the dysregulation of snoRNAs may improve the effectiveness of present clinical drugs or even offer a novel therapeutic approach.

With the development of second-generation high-throughput sequencing technologies, scientists could detect the accurate and specific expression of snoRNAs and their host genes in a variety of hematopoietic diseases. Most of the studies applied high-throughput sequencing methods to profile the expression of snoRNAs and evaluated snoRNAs as a classification and prognostic marker in some diseases. For example, a profile focused on snoRNA expression in acute leukemia found that snoRNAs in the SNORD112-114 cluster were upregulated in APL patients, while most snoRNAs were extensively downregulated in other AML subtypes (41). In addition, in ALK-ALCL (anaplastic large cell lymphoma), snoRNA U3 can distinguish ALK+ from ALK-ALCL samples and might serve as an independent diagnostic marker. A set of snoRNAs, including HBII-239, U59B, and U90, could subdivide AITL/PTCL-NOS patients into three groups (56). Another group performed snoRNA profiling in CLL patients and found that overexpression of twenty sno/scaRNAs was associated with worse treatment-free survival (TFS). CLL patients can be divided into two groups with different progression-free survival (PFS) based on the snoRNA expression profile (72). In summary, the heterogeneity of snoRNAs in/among patients implies that they may be applied in the future diagnosis and prognosis of hematological diseases and might be a powerful tool for evaluating the efficacy of personalized medicine.

## Conclusion and future perspectives

With mounting evidence indicating the implications of snoRNAs in hematopoiesis and hematological malignancies, it is increasingly important to clarify their physio/pathological effects (80). snoRNAs are involved in multiple cellular processes (81–84), including rRNA processing, modification of snRNAs, and modulation of translation and transcription. SNHGs’ effects on tumor repression have been reported previously, albeit their precise roles are still unclear, which may involve the alteration of 2’-O-methylation and pseudouridylation guided by snoRNAs. These alterations have different effects on the initiation and progression of hematological malignancies.

Canonical functions of snoRNAs include guiding chemical modification in rRNA and RNA splicing. In addition, some noncanonical roles of snoRNAs have been discovered recently, such as 2’-O-methylation of tRNA (85). Meanwhile, snoRNA expression is influenced by transcription factors such as c-Myc. Malignant genomic alterations also regulate snoRNA expression. Some unique features of snoRNA expression can help discern the subclassification of hematological malignancies. Although the development of next-generation sequencing (NGS) has facilitated the study of snoRNA function, the bias produced in the process of RNA extraction and library preparation should not be ignored (86–88). To avoid this, there are some new methods applied in RNA purification, for example, isotachophoresis (ITP) (89) or diverse sequencing methods such as AQRNA-seq (90), which could minimize biases and provide more accurate and convincing results. The underlying mechanisms of snoRNAs are still poorly understood and warrant further studies in the future.

SnoRNAs and their host genes exhibit great potential in diagnosis, prognosis, personalized therapy, overcoming drug resistance, and other aspects of clinical application in hematological malignancies (**Supplementary Tables 1** and **2**). Some high-throughput sequencing studies have revealed the involvement of snoRNAs in leukemogenesis and progression (91–93). Further exploration is still needed to develop tools and methods that specifically manipulate snoRNA expression, precisely deliver snoRNAs to disease loci, efficiently minimize side effects and off-target problems.

## Author contributions

PQ supervised the overall project and co-wrote the manuscript. JD, HW wrote the manuscript. ZZ, LY, XQ, WQ, YH, HH contributed to revise and edit of the manuscript. All authors contributed to the article and approved the submitted version.

## Funding

This work was supported by grants from the National Key R&D Program of China, Stem Cell and Translation Research (2018YFA0109300), the National Natural Science Foundation of China (81870080, 91949115, 82161138028, 31900815), the Zhejiang Provincial Natural Science Foundation of China (LR19H080001), the Leading Innovative and Entrepreneur Team Introduction Program of Zhejiang (2020R01006). Thanks for the technical support by the Core Facilities, Zhejiang University School of Medicine.

## Conflict of interest

The authors declare that the research was conducted in the absence of any commercial or financial relationships that could be construed as a potential conflict of interest.

## Publisher’s note

All claims expressed in this article are solely those of the authors and do not necessarily represent those of their affiliated organizations, or those of the publisher, the editors and the reviewers. Any product that may be evaluated in this article, or claim that may be made by its manufacturer, is not guaranteed or endorsed by the publisher.
