# Supplementary material for: Small but strong: Pivotal roles and potential applications of snoRNAs in hematopoietic malignancies
Source: Front Oncol. 2022 Aug 12;12:939465. doi: 10.3389/fonc.2022.939465 (PMC9413531; doi:10.3389/fonc.2022.939465)
Supplement: Supplementary file 1 [file DataSheet_1.pdf]

Table 1. Summary of role and potential clinical application of snoRNA in hematopoietic malignancy

snoRNA	Type	Expression	Disease Subtype	Potential clinical application	Reference
ACA11	orphan	Overexpression	t (4;14)-positive MM	N.A	46
SNORD123	C/D	Overexpression	MM	Prognostic marker	49
SNORD115/SNORD116	C/D	Overexpression	TC2 MM	Subtype marker	47
SCARNA22	scarna	Overexpression	TC4 MM	Pathological marker	47
U75	C/D	Overexpression	ALCL	Subtype marker	57
U3	C/D	Overexpression	ALK+ ALCL	Subtypes marker	57
HBII-239, U59B, U90	C/D	Overexpression	AITC	Prognostic marker	57
SNORD34, SNORD35A, SNORD43	C/D	Overexpression	t (8;21)-positive AML	N.A	44
SNORD42A	C/D	Overexpression	AML	N.A	59
SNORD112-SNORD114	C/D	Downregulation	AML	N.A	42
SNORD112-SNORD114	C/D	Overexpression	APL	Subtype marker	42
SNORA70F, SNORA74	H/ACA	Downregulation	CLL	Prognostic marker	70
SNORD116-118, SNORA74	C/D, H/ACA	Overexpression	CLL	Prognostic marker	70
SNORA12, SNORA22, SNORA27	H/ACA	Downregulation	CLL with IGHV mutation	Prognostic marker	72
SNORD43	C/D	Overexpression	CLL	N.A	73
SNORD24, SNORD44, SNORD82, SNORD105, SCARNA6, SCARNA9	C/D scarna	Overexpression /Downregulation	ALL	Subtype marker	76
SNORD64, SNORD107, SNORD109A, SNORD116	C/D	Overexpression	BCP-ALL with ERG mutations	Subtype marker	77
SNORA7A, SNORA16A, SNORA70	H/ACA	Downregulation	MDS	N.A	80

N.A, not applied.
