# Supplementary material for: Small but strong: Pivotal roles and potential applications of snoRNAs in hematopoietic malignancies
Source: Front Oncol. 2022 Aug 12;12:939465. doi: 10.3389/fonc.2022.939465 (PMC9413531; doi:10.3389/fonc.2022.939465)
Supplement: Supplementary file 2 [file DataSheet_2.pdf]

Table 2. Summary of role and potential clinical application of SNHG in hematopoietic malignancy

SNHG	Expression	Disease Subtype	Potential clinical application	Reference
SNHG18	Overexpression	t (4;14)-positive MM	N.A	49
SNHG12	Overexpression	DLBCL	N.A	50
SNHG14	Overexpression	DLBCL	N.A	52
SNHG16	Overexpression	DLBCL	N.A	53
SNHG16	Overexpression	AML	N.A	62
SNHG5	Overexpression	AML	N.A	63
SNHG1	Overexpression	AML	Prognostic marker	64,65
SNHG4	Downregulation	AML	N.A	66
SNHG5	Overexpression	CML	N.A	68
SNHG5	Downregulation	T-ALL	Therapeutic target	74
SNHG16	Overexpression	ALL	N.A	75

N.A, not applied
